# Atrial Fibrillation and Beta Thalassemia Major: The Predictive Role of the 12-lead Electrocardiogram Analysis

**DOI:** 10.1016/s0972-6292(16)30753-7

**Published:** 2014-05-25

**Authors:** Vincenzo Russo, Anna Rago, Bruno Pannone, Andrea Antonio Papa, Maria Carolina Mayer, Anna Spasiano, Raffaele Calabro, Maria Giovanna Russo, Nigro Gerardo

**Affiliations:** 1 Chair of Cardiology, Second University of Naples, Naples, Italy; 2 Internal Medicine Unit, Cardarelli Hospital, Naples, Italy; 3 Internal Medicine Unit, Cardarelli Hospital, Naples, Italy

**Keywords:** beta-thalassemia major, P-wave dispersion, atrial fibrillation

## Abstract

**Background:**

Paroxysmal atrial tachyarrhythmias frequently occur in beta-thalassemia major (β-TM) patients.The aim of our study was to investigate the role of maximum P-wave duration (P max) and dispersion (PD), calculated trough a new manually performed measurement with the use of computer software from all 12-ECG-leads,as predictors of atrial-fibrillation (AF) in β-TM patients with conserved systolic or diastolic cardiac function during a twelve-months follow-up.

**Materials and Methods:**

50 β-TM-patients (age38.4±10.1; 38M) and 50-healthy subjects used as controls, matched for age and gender, were studied for the occurrence of atrial arrhythmias during a 1-year follow-up, through ECG-Holter-monitoring performed every three months. The β-TM-patients were divided into two groups according to number and complexity of premature-supraventricular-complexes at the Holter-Monitoring (Group1: <30/h and no repetitive forms, n:35; Group2: >30/h or couplets, or run of supraventricular tachycardia and AF, n:15).

**Results:**

Compared to the healthy control-group, β-TM patients presented increased P-max (107.5± 21.2 vs 92.1±11ms, P=0.03) and PD-values (41.2±13 vs 25.1±5 ms,P=0.03). In the β-TM population, the Group2 showed a statistically significant increase in PD (42.8±8.6 vs 33.2±6.5ms, P<0.001) and P-max (118.1±8.7 vs 103.1±7.5ms, P<0.001) compared to the Group1. Seven β-TM patients who showed paroxysmal AF during this study had significantly increased P-max and PD than the other patients of the Group2. Moreover, P-max (OR:2.01; CI:1.12-3.59; P=0.01) and PD (OR=2.06;CI:1.17-3.64;P=0.01) demonstrated a statistically significant association with the occurrence of paroxysmal AF,P min was not associated with AF-risk (OR=0.99; CI:0.25-3.40; P=0.9) in β-TM-patients. A cut-off value of 111ms for P-max had a sensitivity of 80% and a specificity of 87%, a cut-off value of 35.5ms for PD had a sensitivity of 90% and a specificity of 85% in identifying β-TM patients at risk for AF.

**Conclusion:**

Our results indicate that P-max and PD are useful electrocardiographic markers for identifying the β-TM-high-risk patients for AF onset, even when the cardiac function is conserved.

## Introduction

Beta-Thalassemia Major (β-TM) is a hereditary haemoglobin disorder caused by reduced synthesis of β-globin chain and resulting in a chronic haemolytic anaemia. The consequences of chronic anaemia include growth retardation, bone marrow expansion, extramedullary hematopoiesis, splenomegaly, greater intestinal iron absorption, hypercoagulability and higher susceptibility to infections [1]. In the early stage, patients are usually asymptomatic. Restrictive cardiomyopathy usually occurs before dilated cardiomyopathy [2], in accordance with diastolic dysfunction which normally happens before systolic dysfunction and overt heart failure [3,5]. Findings in the early stage are usually incidental, including bradycardia, ST-T changes, infrequent premature atrial or ventricular contractions, first-degree atrioventricular blocks and evidence of left ventricular hypertrophy. In the late stage, frequent premature atrial or ventricular contractions, runs of supraventricular tachycardia, atrial flutter and fibrillation, ventricular tachycardia and second-degree or complete heart blocks have been demonstrated [2,5,6].

QTc and JTc dispersion are useful electrocardiographic markers of sudden cardiac death risk and are increased in some conditions [7-12]. Our previous study suggested that beta thalassemia major is associated with significant changes in heterogeneity of ventricular repolarization, the electrophysiological substrate for ventricular malignant tachyarrhythmias, and the use of QTc dispersion and JTc dispersion, as simple electrocardiographic parameters for stratifying the sudden cardiac death risk in beta thalassemia major patients, should be implemented in our daily clinical practice [13].

The identification of β-TM patients at risk for atrial fibrillation is of pivotal importance for the optimization of the medical therapy to prevent tromboembolic stroke. Maximum P wave duration (P max) and P wave dispersion (PD) are two simple electrocardiographic markers considered to reflect the discontinuous and inhomogeneous propagation of sinus impulses and the prolongation of atrial conduction time [14,15]. PD was shown to be an independent risk factor for development of atrial fibrillation [16,17]. In a previous study [18], we showed a significant increase of P wave dispersion, correlated to myocardial iron deposit, assessed by Cardiac Magnetic Resonance (CMR) T2 imaging, in β-TM patients with conserved systolic and diastolic cardiac function. However, to our knowledge, there are no studies evaluating the predictive value of PD and P max on new onset AF occurrence in β-TM patients.

The aim of the present study was to determine whether maximum P-wave duration and P-wave dispersion detected on surface ECG could predict the new onset paroxysmal atrial fibrillation occurrence in β-TM patients with conserved systolic and diastolic cardiac function.

## Materials and Methods

### Study population

The study involved 50 β-TM subjects (38 men, 12 women), with a mean age of 38.4 ± 10.1 years and mean body mass index (BMI) of 20.1 ± 3.1 kg/m2. All patients were consecutively recruited from the Internal Medicine of Cardarelli Hospital to participate in the study and followed for a 12 months period. Fifty sex and age-matched non β-TM healthy subjects were also recruited as controls. Inclusion criteria were age between 18 and 50 years. Subjects with a history of hypertension (systolic and diastolic blood pressure < 140/90 mmHg), diabetes mellitus or impaired glucose tolerance (IGT), obesity, electrolyte imbalance, valvular heart disease, heart failure, coronary artery disease, systolic and diastolic dysfunction, connective tissue disorders, left bundle branch block or atrioventricular conduction abnormalities on electrocardiogram (ECG), previous AF episodes, hepatic, renal, thyroid diseases, sleep disorders, or having a permanent pacemaker were excluded from the study. All patients were in sinus rhythm, and none of them was taking medications known to affect electrocardiographic intervals (antiarrhythmic agents, tricyclic antidepressants, antihistaminics, or antipsychotics). All patients were requiring regular blood transfusions at 2-4 weekly intervals to maintain haemoglobin levels over 9 g/dl. The patients were receiving iron chelation therapy either as standard desferrioxamine (DFO) 40-50 mg/kg sc 5 days a week or as deferiprone (DFP), which was given at a daily dose of 75 mg/kg either as a single agent or in combination with DFO (40-50 mg/kg twice weekly).

### Study protocol

Medical history, physical examination, anthropometric evaluation, 12-lead surface ECG, ECG Holter monitoring and 2D color doppler echocardiogram were performed in the study population. The cardiac evaluation was standardized to blood transfusion interval and was performed at a median of 5 days (ranged 1-8) following transfusions and every three months during the 12 months follow up. The patients were rested for at least 15 min before cardiovascular assessments, including electrocardiography and echocardiography. All patients gave their informed consent.

### Electrocardiographic measurements

All subjects underwent a routine standard 12-lead body surface ECG recorded at a paper speed of 50 mm/s and gain of 10 mm/mV in the supine position and were breathing freely but not allowed to speak during the ECG recording. To avoid diurnal variations, we generally evaluated the ECG recordings at the same time (9:00-10:00 A.M.). The analysis was performed by one investigator only without knowledge of subject's clinic status. ECGs were transferred to a personal computer by an optical scanner and then magnified to 400% by Adobe Photoshop software (Adobe Systems Inc., San Jose, CA). P-wave duration measurement was manually performed with the use of computer software (Configurable Measurement System) from all 12 ECG leads. These new PD measurement method allowed us to minimize measurement errors done with manual evaluation that may be a potential bias for observed results and to overcome the limitations of the measurement of a useful electrocardiographic parameter as PD, the validity of which has been much debated in the literature. Intra-observer coefficients of variation for P wave variables were found to be less than 5% and not significant. In each electrocardiogram lead, the analysis included three consecutive heart cycles wherever possible. ECG with measurable P-wave in less than ten-leads were excluded from analysis. The onset of P wave was defined as the junction between the isoelectric line and the start of P-wave deflection; the offset of the P-wave as the junction between the end of the P-wave deflection and the isoelectric line [19,20]. If starting points and endpoints were not clear, the derivations including these points were taken as excluding criteria from the study. Maximum and minimum P-wave durations were measured. Maximum P-wave duration was defined as the longest P-wave duration, and minimum P-wave duration was defined as the shortest P-wave duration. P-wave dispersion was defined as the difference between the maximum P-wave duration and the minimum P-wave duration.

### Echocardiographic measurements

Images were gathered with a standard ultrasound machine with a 3.5-MHz phased-array probe (M3S). All the echocardiographic studies were digitally stored and all the measurements were performed off-line by two independent observers who were blinded to the clinical status of the subjects. Left ventricular (LV) diameter and wall thickness were measured from the two dimensional targeted M-mode echocardiographic tracings in the parasternal short axis. Ejection fraction was measured using a modified Simpson's biplane method. Each representative value was obtained from the average of three measurements. LV mass was determined and indexed to height (meters) to the power of 2.7 (LVM/H 2.7). Pulsed-wave Doppler examination was performed to obtain the following indexes of LV diastolic function: peak mitral inflow velocities at early (E) and late (A) diastole and E/A ratio. Average values of these indexes obtained from 5 consecutive cardiac cycles were used for analysis.

### Holter recording

Seven leads 24h ECG Holter recording was performed using a commercially available equipment (Cardioscan Holter System, DMS, Stateline NV, USA) every three months during the 12 months follow up period. The β-TM patients were divided into two groups according to number and complexity of supraventricular arrhythmias at the Holter Monitoring: Group 1: <30/h premature supraventicular complexes (PSCs) and no repetitive forms (n: 35 patients); Group 2: >30/h premature supraventicular complexes or supraventricular couplets or supraventricular tachycardia and atrial fibrillation (n: 15 patients). All healthy control subjects underwent 24 h ECG Holter monitoring every three months during the observation period and they did not show atrial or ventricular arrhythmias (they presented less than 10 premature supraventricular or ventricular complexes during 24 hours at the ECG Holter Monitoring).

### Statistical Analysis

Continuous variables were expressed as mean values ± standard deviation (SD). Statistical analysis was performed using Student's t-test. Multivariable analysis with a Cox proportional hazards model was used to test whether the occurrence of paroxysmal AF was related to age, gender, PD and P max. A receiver-operating characteristics (ROC) curve was constructed to identify the P wave cutoff values that differentiate patients with and without occurrence of paroxysmal AF with the best sensitivity and specificity levels. In all statistical tests, calculated P values of less than 0.05 were considered statistically significant. Correlations were performed using Spearman's correlation analysis. Statistical comparisons were performed using the statistical software package SPSS 10.01 (SPSS Inc., Chicago, IL, USA).

## Results

### Clinical and echocardiographic parameters

Table 1 summarizes the clinical, laboratory and echocardiographic characteristics of the study population. Healthy control group did not significantly differ from β-TM group in BMI, heart rate, blood pressure (BP) and atrial diameters and areas. The β-TM group showed significantly higher left ventricular posterior wall end diastolic thickness (LVPWEDT: 9.1 ± 0.4 vs 6.7 ± 1.8 mm; P = 0.04), inter- ventricular septum end diastolic diameter thickness (IVSEDT: 9.3 ± 0.9 vs 6.1 ± 1.1 mm; P = 0.04), left ventricular end diastolic diameter (LVEDD: 53.8 ± 4.9 vs 42.2 ± 3.1 mm; P = 0.04), left ventricular end systolic diameter (LVESD: 33.5 ± 5.6 vs 25.3 ± 2.6 mm; P = 0.04). However, no significant difference in left ventricle fractional shortening (FS: 31.8 ± 4.1 vs 33.8 ± 3.9 %; P = 0.3) and ejection fraction (EF: 64.3 ± 7.9 vs 65.1 ± 4.9 %; P = 0.2) between the two groups were observed. These data indicate compensated normal systolic function in the β-TM group. Compared with controls, the β-TM group did not show significantly E wave (81.8 ± 16.4 vs 91.3 ± 10.6 cm/s; P = 0.3), A wave (58.8 ± 13.1 vs 51.1 ± 8.9 cm/s; P = 0.2) and E/A ratio (1.4 ± 0.5 vs 1.6 ± 0.4; P = 0.2) variations. These data indicate normal diastolic function in the β-TM group.

### P wave duration and dispersion

Electrocardiographic characteristics of the study population are shown in Table 2. Compared with healthy control group, thalassemic patients presented increased maximum P wave duration (107.5 ± 21.2 vs 92.1 ± 11 ms, P = 0.03) and P-wave dispersion values (41.2 ± 13 vs 25.1 ± 5 ms, P = 0.003). No statistically significant difference was found in heart rate (76.8 ± 5.4 vs 75.7 ± 6.3 bpm, P= 0.3), PR interval (148 ± 13 vs 142 ± 8 ms, P= 0.4) and minimum P wave duration (68.6 ± 12 vs 66.2 ± 7 ms, P = 0.4).

### Maximum P-wave duration, P wave dispersion and atrial fibrillation incidence

**Figure 1:**
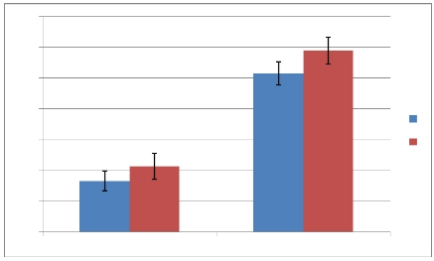
P wave dispersion (PD) and maximum P wave duration (P max) in the two groups of β-TM patients separated according to the ECG Holter monitoring results (Group 1: <30/h and no repetitive forms, n: 35; Group 2: >30/h or couplets, or run of supraventricular tachycardia and atrial fibrillation, n: 15)

In the β-TM population, the Group 2 showed a statistically significant increase in PD (42.8 ± 8.6 vs 33.2 ± 6.5 ms, P< 0,001) and P max (118.1 ± 8.7 vs 103.1 ± 7.5 ms, P< 0,001) compared to the Group 1 (Figure 1). No statistically significant difference was shown in HR (77.8 ± 5.3 vs 84.9 ± 4.5 beats/min; P= 0.3), PR interval (159 ± 10 vs 143 ± 8 ms, P=0.4) and P min (72.9 ± 7.1 vs 74.3 ± 3.6 ms, P= 0,16). Seven β-TM patients who showed asymptomatic paroxysmal atrial fibrillation (mean AF episodes number: 30.1± 12; mean AF episodes duration: 38.4 ± 11 minutes; mean AF episodes heart rate: 110.5 ± 26 bpm) during this study had significantly increased P max (119.7 ± 8.3 vs 105.1 ± 7.6 ms, P < 0.001) and PD (45.1 ± 8.4 vs 35.2 ± 6.5 ms, P < 0.001) than the other members of the Group 2. A cut off value of 111 ms for P max had a sensitivity of 80% and a specificity of 87% in identifying β-TM patients at risk for atrial fibrillation, with a positive predictive value of 50% and a negative predictive value of 96%. A cut off value of 35.5 ms for PD had a sensitivity of 90% and a specificity of 85% in identifying this category of patients, with a positive predictive value of 50% and a negative predictive value of 98%. The incidence of AF was 2% (1/50) among patients with P wave dispersion ≤ 30 ms, 4% (2/50) in patients with P wave dispersion > 30 and ≤ 35.5 ms, and 8% (4/50) in patients with P wave dispersion more than 35.5 ms. P min did not achieve statistical significance in multivariable logistic model (OR = 0.99; CI: 0.25-3.40; P = 0.9), whereas P max (OR:2.01; CI:1.12-3.59; P = 0.01) and PD (OR = 2.06; CI: 1.17-3.64; P = 0.01) were significantly associated with AF risk. However, using the multivariable analysis, only PD ≥ 35.5 ms (P= 0.04) remained significant as independent predictor of occurrence of AF in the study population (Table 3).

### Association between AF and age or gender

In the multivariable logistic regression model, younger age was associated with a higher AF risk, but this trend was not statistically significant (OR = 1.21; CI: 0.53-2.72; P = 0.6). As regard gender, males showed a greater AF risk with respect to females, but also in this case statistical significance was not reached (OR = 2.27; CI: 0.26-18.96; p = 0.4).

## Discussion

Heart complications represent the leading cause of mortality in thalassemia major, even though, following the introduction of chelating therapies, an important and progressive increase of life expectancy has been demonstrated [21]. While iron-induced cardiomyopathy is slowly progressive and it usually takes several decades for clinical or laboratory features of cardiac dysfunction to manifest, atrial fibrillation may be present also if only isolated myocardial siderosis, without signs of cardiac dysfunction, is present. The risk of stroke/embolism is increased 5-fold in patients with AF compared to subjects in sinus rhythm. The cardio-embolic stroke has been reported in 0.25-0.46% of patients with β-TM in different endemic countries [22,23]. While patients with sickle - β thalassemia and thalassemia intermedia present asymptomatic ischaemic lesions that spare the cortex [24,25], patients with β-TM seem to suffer large hemispheric territorial infarcts in presence of AF and cardiomyopathy [26]. No data are currently available regarding the prophylactic efficacy of antiplatelet or anticoagulant drugs for prevention of thromboembolism related to atrial fibrillation in β-TM population, and this now requires dedicated studies. The 12-lead resting ECG remains the most frequently used examination in the evaluation of patients for cardiovascular disease and, because of its relatively low cost, it has the greatest potential to be used as a screening tool. The ECG ability to predict AF may identify a group of beta thalassemia patients whose thromboembolic stroke risk can be modified. To our knowledge, there are no studies evaluating the predictive value of PD and P max on AF recurrence in β-TM patients. The aim of the present study was to determine whether maximum P-wave duration and P-wave dispersion detected on surface ECG may could predict the new onset paroxysmal atrial fibrillation occurrence in β-TM patients with conserved systolic and diastolic cardiac function.

### P wave dispersion and chronic anaemia

Little is known in literature about the P wave dispersion in chronic anaemia patients. Simsek et al. [27] showed that P max and PD were significantly prolonged in 97 patients with iron deficiency anemia and mean haemoglobin value of 7.9±1.6 g/dl; they hypothesized that compromised oxygen delivery capacity due to anaemia may result in chronic tissue hypoxemia, which may then lead to myocyte dysfunction.

In our previous study, we investigated the P-wave dispersion in beta thalassemia patients with normal systolic and diastolic function and correlated the PD to myocardial iron deposit, assessed by CMR T2* imaging. Our data showed a significant increase in P max and PD in β-TM patients with conserved systolic and diastolic cardiac function, compared to sex and age-matched normal controls, and suggested the hypothesis that iron overload toxicity per se influences earlier the propagation of sinus impulses and the atrial conduction time than mechanical cardiac function [18].

### P wave dispersion and atrial fibrillation

P wave dispersion is a non invasive indicator of intra-atrial conduction heterogeneity producing substrate for reentry, which is one of pathophysiological mechanisms of atrial fibrillation [28]. PD has been studied in some other conditions such as hypertension [29], obesity [30], diabetes mellitus [31], metabolic syndrome [32], dilated cardiomyopathy [33], myocardial infarction [34], atrial septal defect [35], hypertrophic cardiomyopathy [36], obstructive sleep apnea [37], Emery Dreifuss muscular dystrophy [38] and Wilson's disease [39]. The exact mechanism of PD prolongation in these clinical conditions is not well known, but it is thought that structural and electrophysiological changes in the atrial myocardium caused by elevated plasma volume [40], ventricular diastolic dysfunction [41] and enhanced neurohormonal activation [42], typical conditions of these deseases, may contribute to left atrial enlargement and electrical instability. Dilaveris et al. [43]. firstly showed that prolonged P wave duration and P wave dispersion may be used as predictors of frequently relapsing AF. In a large cohort of patients, Perez et al. [44] confirmed prior observations that P wave duration, P wave dispersion, abnormal P axis and left atrial enlargement were predictive of AF and introduced the P wave index, defined as the SD of P-wave duration across the 12 leads, as novel measurement that could better represent the atrial heterogeneity. According to their findings, P wave index > 35 was one of the strongest predictors of AF (hazard ratio: 2,7). There is no consensus about the cutoff value for PD that separates patients who have a history of paroxysmal AF from healthy subjects. Dilaveris et al. identified 40 ms as cutoff value of PD to separate patients with paroxysmal AF from controls, while Aytemir et al. [45] used a PD cut off value of 32.5 ms in another study, which excluded patients with structural heart disease. The differences in the cutoff values between these studies may be due to the different patients' clinical characteristics and measurement methods used.

### Main Findings

Studying the effect of beta thalassemia major in patients without systolic or diastolic dysfunction and without other clinically appreciable cause of heart, hepatic, renal, thyroid and metabolic diseases, might have offered the unique clinical opportunity to exclude the influence of possible comorbidities on the evaluation of inhomogeneous propagation of sinus impulses and the relationship with the onset of atrial fibrillation. Our data confirmed that the electrocardiographic parameters proposed to estimate the discontinuous and inhomogeneous propagation of sinus impulses and the prolongation of atrial conduction time (Pmax and PD) were significantly increased in β-TM patients when compared with age and sex-matched healthy controls. We showed a statistically significant increase in PD and P max in beta thalassemia major patients at high risk for atrial fibrillation onset and we suggested the hypothesis that the abnormal P wave dispersion ≥35.5 ms and maximum P wave duration ≥111 ms may predict atrial fibrillation onset in beta thalassemia major patients with conserved systolic and diastolic function.

### Limitations

The small number of patients included is certainly a limitation and a more extensive study is needed to confirm these data. P wave dispersion measurement errors done with manual evaluation may be a potential bias for observed results, although, according to Dilaveris et al. [35], scanning and digitizing ECG signals from paper records using an optical scanner is a feasible and accurate method for measuring P wave duration. No difference was showed in systolic and diastolic function in the study population, however the β-TM group showed a statistically significant difference in ventricle's wall diameter and thickness respect to the sex and age-matched healthy control group. These differences indicate that the cardiac structure in thalassemia patients may not be completely normal, even when the systolic and diastolic function is preserved. The relationship between P wave dispersion, cellular cardiac electrophysiological properties and cardiac structural changes are complex and still waiting for investigation on a larger study population.

## Conclusion

Our study demonstrated that P wave dispersion measurement seems to be a fairly good marker for identifying the β-TM high-risk group for atrial fibrillation onset, even when the cardiac function is conserved. Our data suggest that the use of P wave dispersion, as simple electrocardiographic parameter for the atrial fibrillation risk assessment in beta thalassemia major patients, should be implemented in our daily clinical practice. PD cut off value ≥ 35.5 ms and P max cut off value ≥ 111 ms may identify high risk atrial fibrillation β-TM patients who need a careful cardiac monitoring. For these patients, we suggest seriate ECG Holter recordings to early detect atrial fibrillation onset and to evaluate the opportunity of prophylactic anticoagulation treatment.

## Figures and Tables

**Table 1:**
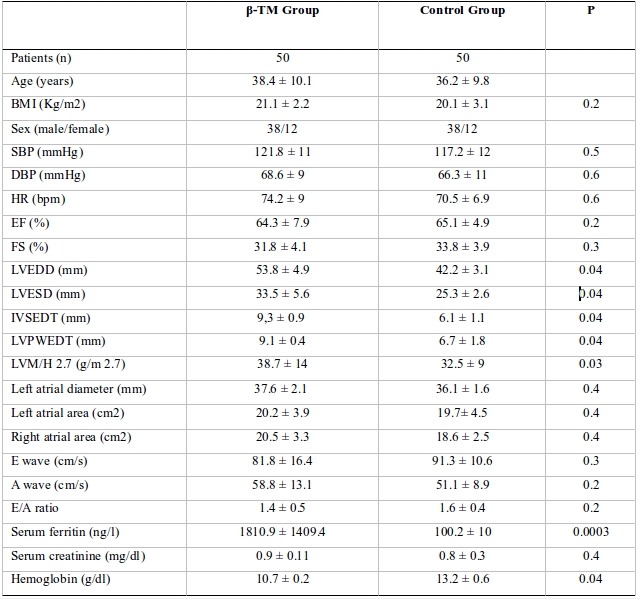
Clinical, laboratory and echocardiographic characteristics of the study population

BMI: body mass index; SBP: systolic blood pressure; DBP: diastolic blood pressure; HR: heart rate; EF: ejection fraction; SF: shortening fraction; LVEDD: left ventricular end diastolic diameter; LVESD: left ventricular end systolic diameter; IVSEDT: interventricular septal end diastolic diameter; LVPWEDT: left ventricular posterior wall end diastolic thickness; LVM/H: left ventricular mass/height

**Table 2:**
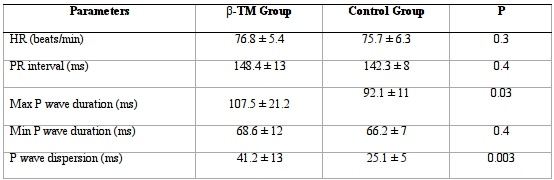
Electrocardiographic characteristics of the study population

HR: heart rate; PR: atrioventricular interval

**Table 3:**
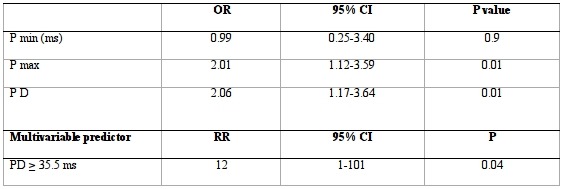
Results at multivariable analysis of P-wave characteristics and multivariable predictor of occurrence of AF in β-TM patients
