# Atrial Fibrillation and Beta Thalassemia Major: The Predictive Role of the 12-lead Electrocardiogram Analysis

**DOI:** 10.1016/s0972-6292(16)30801-4

**Published:** 2014-10-06

Name of last author has been corrected as: Gerardo Nigro

